# Susceptibility of Foodborne Pathogens to Milk-Origin Lactic Acid Bacteria Supernatants: A Comprehensive Meta-Regression Study

**DOI:** 10.3390/foods13162635

**Published:** 2024-08-22

**Authors:** Nathália Fernandes, Yara Loforte, Vasco Cadavez, Ursula Gonzales-Barron

**Affiliations:** 1 Centro de Investigação de Montanha (CIMO), Instituto Politécnico de Bragança, Campus de Santa Apolónia, 5300-253 Bragança, Portugal; nathalia@ipb.pt (N.F.); yara_loforte@hotmail.com (Y.L.); vcadavez@ipb.pt (V.C.); 2 Laboratório para a Sustentabilidade e Tecnologia em Regiões de Montanha, Instituto Politécnico de Bragança, Campus de Santa Apolónia, 5300-253 Bragança, Portugal; 3 Divisão de Agricultura, Instituto Superior Politécnico de Manica, Campus de Matsinho, Manica 417, Mozambique

**Keywords:** antimicrobial peptides, hurdle technology, cheese, lactic acid bacteria

## Abstract

This systematic review and meta-analysis compile the in vitro antimicrobial efficacy of lactic acid bacteria (LAB) supernatants against three common pathogenic bacteria found in dairy products: *Salmonella* spp., *L. monocytogenes*, and *Staphylococcus aureus*. After screening and analysis of full papers, identified by searches in PubMed, Scopus, and Web of Science databases, thirty-nine studies were regarded as relevant, and a total of 510 observations were recorded. The effects of moderators on inhibition diameters were assessed by adjusting three pathogen-specific meta-regression models. Results showed that, in general terms, strains from the *Enterococcus* genus displayed the highest inhibition values against *L. monocytogenes* (15.90 ± 2.138 mm), whereas *Lacticaseibacillus* strains were more effective against *S. aureus* (11.89 ± 0.573 mm). The well diffusion test outperformed the spot and disk diffusion tests, and more acidic LAB supernatants resulted in higher measurements of inhibition diameters (*p* < 0.001). Meta-regression models incorporating LAB genus, pathogen concentration, and incubation time explained 33.8%, 52.3%, and 19.8% of the total variance in inhibition diameters for *L. monocytogenes, Salmonella* spp., and *S. aureus*, respectively. None of the three models showed evidence of publication bias. This meta-regression study demonstrated that LAB strains present in dairy products possess a variable capacity to inhibit any of the three foodborne pathogens. Overall, *L. monocytogenes* was found to exhibit greater susceptibility than *Salmonella* spp. and *S. aureus*; thus, the antilisterial capacity of the selected LAB strains could be exploited in developing biocontrol strategies for cheese-making.

## 1. Introduction

Fermented milk products such as cheese have a long history of preservation and culinary significance. In 2022, the European Union (EU) produced 160 million tons (m.t.) of raw milk, predominantly sourced from bovine (154.3 m.t.), ovine (3 m.t.), caprine (2.5 m.t.) and bubaline (0.3 m.t.) origins [1]. A portion of this raw milk was converted into cheese (10.4 m.t. of total production), with artisanal cheese production, particularly thriving in the Mediterranean regions [2,3,4]. However, artisanal cheese-making operations face challenges related to microbial contamination. To address this issue, there is a growing trend toward employing natural lactic acid bacteria (LAB) cultures to mitigate contamination risks and ensure the microbial safety and quality of cheese [3,4].

LAB constitute a specific taxonomic group of microorganisms characterized by their Gram-positive nature, lack of catalase enzyme, and limited mobility [5]. Owing to their technological and probiotic potential, autochthonous LAB have been sought [6]. Artisanal cheeses, particularly those made from raw milk, are rich sources of microbial diversity, especially for the discovery of new LAB strains. During fermentation, LAB produce substances that prevent harmful bacteria from growing in food. The main mechanism to achieve this is by producing lactic acid, which lowers pH and negatively influences bacteria survival [7]. LAB also releases other compounds, such as hydrogen peroxide, carbon dioxide, diacetyl, and bacteriocins, which further prevent bacterial growth [5].

However, ensuring the safety of raw milk cheese relies on various factors, including the type of cheese, initial microbial contamination, and the presence of harmful pathogens [6]. Foodborne diseases, as defined by the World Health Organization (WHO), are illnesses caused by consuming food contaminated with harmful microorganisms and their toxins [8,9]. These diseases can lead to a range of severe symptoms, including organ failure, digestive issues, immune system problems, and neurological complications [9,10].

Contaminated food poses a significant threat to both food safety and human health. There have been 181 notifications since 2020 for milk and milk products in the EU, according to the Rapid Alert System for Food and Feed (RASFF) [11]. Pathogens such as *Listeria monocytogenes* (n = 106), *Salmonella* spp. (n = 15), and *Staphylococcus aureus* (n = 3) are frequently associated with outbreaks of cheese consumption (124 of 181 notifications) [10,11]. 

Given the association of milk and its derivatives with various pathogenic microorganisms, improving the microbiological quality of cheese is imperative, warranting intensified efforts to ensure the safety of these products [12]. Traditional cheeses made from raw milk, without added starter cultures, harbor indigenous LAB strains with protective properties against pathogenic bacteria and are more suited for cheese-making environments compared to commercial starters [13]. Moreover, using LAB for natural food preservation meets the demand for healthier foods with fewer chemical additives and contributes to expanding the range of functional dairy products [14,15].

Numerous studies have focused on isolating autochthonous LAB strains and testing their efficacy against common pathogens. However, the lack of standardization and the absence of a unified protocol for evaluating these traits pose challenges. The variability in methodologies across studies makes it difficult to compare the results and draw comprehensive conclusions. 

This study aimed to (1) perform a systematic review and meta-analysis of the in vitro susceptibility of foodborne pathogens, namely, *L. monocytogenes*, *Salmonella* spp., and *S. aureus*, to autochthonous LAB species isolated from dairy products and (2) assess the relative importance of different moderators in LAB supernatant antimicrobial activity. To achieve this, separate multilevel meta-analyses were conducted using up-to-date information extracted from relevant literature sources.

## 2. Materials and Methods

This systematic review was conducted in accordance with the Preferred Reporting Items for Systematic Reviews and Meta-Analyses (PRISMA) guidelines, which aim to improve the reporting of systematic reviews and meta-analyses, with the following statement questions: “Which LAB genus display the highest levels of antimicrobial activity; and which moderators influence the measured antimicrobial activity of LAB supernatant against pathogenic bacteria?” [16].

### 2.1. Bibliographic Search

Electronic searches were conducted in the PubMed, Scopus, and Web of Science databases to gather articles presenting results from in vitro assays carried out with autochthonous dairy LAB and their antimicrobial effects against foodborne pathogens such as *L. monocytogenes*, *Salmonella* spp., and *S. aureus*. The search was carried out by two reviewers in November 2022. The abstract bibliographic search was defined by terms encompassing cheese, starter culture, and antimicrobial properties against specific pathogens as follows:

(cheese OR milk OR dairy) AND (lactic acid bacteria OR starter culture OR biocontrol OR *lactobacillus*) AND (antimicrobial* OR functional OR preserv* OR biopreserv* OR antagoni*) AND (*Salmonella* OR *Listeria monocytogenes* OR *Staphylococcus aureus* OR pathogen*).

Searches were carried out using the specific syntax of PubMed, Scopus, and Web of Science.

### 2.2. Data Extraction

Duplicate references were identified with the Rayyan software [17]; reviewers manually verified and removed duplicates. Subsequently, individual reviewers examined the titles and abstracts of the citations retrieved. The title and abstract evaluation adhered to specific inclusion criteria: (i) the article must be written in English or Portuguese; (ii) the article must be a primary study, this is, must present original results; (iii) studies should present results of inhibition diameter of LAB against at least one of the selected pathogens; (iv) articles must specify the origin of LAB, and it should be of dairy origin; and (v) studies must have LAB identified at species level.

In the second step, the full text of selected articles were assessed to confirm their inclusion, considering any supplementary material if available. If the full text could not be retrieved, the corresponding author was contacted to request access [18]. Additional inclusion criteria for the full-text screening phase included the reporting of inhibition diameter or radius in continuous numeric values. Furthermore, the article had to provide the sample size or number of repetitions (N).

Next, data extraction was performed from the selected articles using Excel [19]. The extracted information encompassed details regarding the origin of LAB (milk source [cow, goat, sheep, NA], food type [milk, cheese, yogurt]), identification of LAB and pathogens (genus, species and strain), parameters from the susceptibility test method [agar spot, disc diffusion, well diffusion], duration of incubation, temperature of incubation, agar medium [Muller-Hinton, De Man–Rogosa–Sharpe, Nutrient, Trypticase soy, Brain Heart Infusion], mode of LAB application (bacteriocin or supernatant) and variations in protocol: concentration of pathogen, concentration of LAB, aliquot volume, pH of LAB (bacteriocin or supernatant).

### 2.3. Meta-Regression Modeling

This meta-analysis considered LAB to be evaluated for their antimicrobial activity as the study population. The primary measured outcome was the mean inhibition diameter (ID) in millimeters, which refers to the total halo formed around the colony, including the measurement of the colony, well, or disk in the final ID calculation [20]. If the articles presented the results in terms of radius, the values were converted to diameters.

Two types of multilevel random-effects meta-analysis regressions were fitted to data in order to answer different questions: the overarching meta-regression and the pathogen-specific meta-regressions.

#### 2.3.1. Overarching Meta-Regression Models

The meta-regression model of the type,

(1) $${ID}_{pgaj}=\left(\beta_{0}+u_{j}\right)+\beta_{1}\cdot {Pathogen}_{p}+\beta_{2}\cdot {LABgenus}_{g}+{\beta_{3}\cdot AntagonismAssay_{a}+\beta }_{4}\cdot [Pathogen]\;+\beta_{5}\cdot IncubationTime+\beta_{6}\cdot AliquotVolume+\epsilon_{pgaj}$$

was adjusted to the full data, where *β*_0_ is an intercept, and *β*_1_, *β*_2,_ and *β*_3_ are sets of fixed effects of the *p* types of pathogens, the *g* types of LAB genus, and *a* types of antagonism assay methods. The term *β*_4_ is the effect of a one log increase in pathogen concentration (log CFU/mL); *β*_5_ the effect of the incubation time (h), and *β*_6_ the effect of the aliquot volume (μL). The error term *ε_pgaj_* represents the residuals and accounts for the variability between studies *j*. The remaining unexplained variability was extracted by placing random effects *u_j_* due to study *j* in the intercept *β*_0_.

The purpose of this model was to compare the extent of susceptibility to LAB supernatant among the three pathogens. Next, by removing the pathogen effect *β*_1_ from Equation (1), the same model was fitted to data subsets partitioned by the type of pathogen. The purpose of adjusting these three other models was to obtain meta-analytical estimates of ID (pooled ID) by LAB genus and susceptibility method, solved for a pathogen concentration of 7.0 log CFU/mL and an incubation time of 24 h. In this way, the pooled ID aligned under the same conditions allowed for comparisons between the pooled estimates.

#### 2.3.2. Pathogen-Specific Meta-Regression Models

Three separate meta-regression models were adjusted for pathogens. Data were partitioned by *L. monocytogenes*, *Salmonella* spp., and *S. aureus*, and the following models were adjusted, respectively,

(2) $${ID}_{gj}=\left(\beta_{0}+u_{j}\right)+\beta_{1}\cdot {LABgenus}_{g}+\beta_{2}\cdot [Pathogen]+\beta_{3}\cdot IncubationTime+\epsilon_{gj}$$

(3) $${ID}_{amj}=\left(\beta_{0}+u_{j}\right)+\beta_{3}\cdot AntagonismAssay_{a}+\beta_{2}\cdot {Agar}_{m}+\beta_{3}\cdot IncubationTime+\epsilon_{amj}$$

(4) $${ID}_{agj}=\left(\beta_{0}+u_{j}\right)+\beta_{1}\cdot {LABgenus}_{g}+\beta_{2}\cdot [Pathogen]+\beta_{3}\cdot AntagonismAssay_{a}+\epsilon_{agj}$$

where the coefficients can be interpreted as exposed beforehand; and, in addition, in Equation (3), *β*_2_ is the set of fixed effects for the types of agar. It can be observed that a different meta-regression solution was achieved for each pathogen. This occurs because the partitions have different data structures, which is very common in meta-analytical data sets; moreover, the models are data-driven. For the three pathogen-specific models, only significant terms were retained. Non-significant terms were dropped at α = 0.10. The purpose of the pathogen-specific meta-regressions was to assess the effect of moderators and, therefore, deduce which moderators are more determinant in observing changes in the measured ID. To further understand the effect of certain quantitative moderators, bubble plots were generated. Meta-analysis models and graphs were built in R version 4.3.3 [21] using the metafor package [22].

### 2.4. Assessment of Heterogeneity and Publication Bias

Assessment of heterogeneity and publication bias were undertaken for each of the three pathogen-specific meta-regressions. First, from the null model (intercept only), within-study variability (*s*^2^) and between-study variability (*τ*^2^) were obtained. The intra-class correlation (*I*^2^) of the null model was then calculated as,

(5) $$I^{2}=\frac{\tau^{2}}{\left(\tau^{2}+s^{2}\right)}\times 100\%$$

The *I*^2^ statistic measures the percentage of variation due to between-study heterogeneity, with values below 25% indicating low heterogeneity, between 25 and 50% moderate, and above 50% high heterogeneity. As a rule of thumb, if *I*^2^ > 25%, an attempt should be made to explain the variability between studies by incorporating significant moderators (also known as study characteristics). After fitting the full (final) model, the residual between-study variability (*τ*^2^*_res_*) is obtained. Next, the between-study variability explained by significant moderators (*R*^2^) can be estimated as,

(6) $$R^{2}=\frac{\left(\tau^{2}-\tau_{res}^{2}\right)}{\tau^{2}}$$

Publication bias was ascertained using two methods: (1) by adding the sample size N as a moderator to each of the pathogen-specific models (Equations (2)–(4)) and applying the decision rule that publication bias is likely if *p* < 0.05; and (2) by constructing funnel plots to visually identify potential publication bias and further evaluate the heterogeneity within a meta-regression based on the spread of standard errors among individual outcomes [23].

## 3. Results

### 3.1. Description of the Meta-Analytical Data

**Figure 1:**
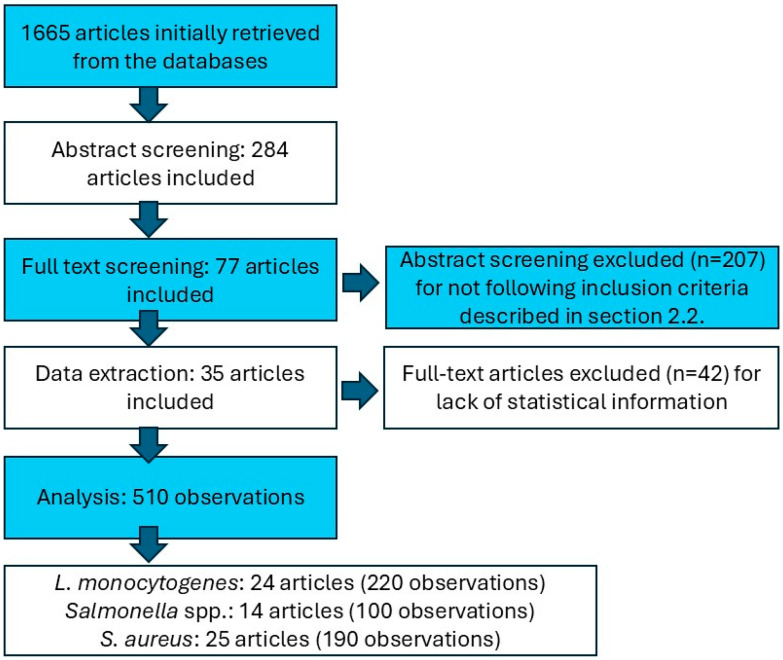
PRISMA flow diagram.

The study selection process is illustrated in the PRISMA flow diagram (Figure 1). A total of 1665 articles were initially retrieved from the databases. Following the removal of duplicates and abstract screening, two hundred and eighty-four articles remained. Subsequently, seventy-seven articles were included after the full-text screening phase. Of these, thirty-five primary studies met the quality inclusion criteria, presenting sufficient data in an extractable form. This resulted in 510 observations to be analyzed. The analysis of the studies identified eight studies conducted prior to 2015 and 27 after 2015. The meta-analyses for each pathogen subset, such as *L. monocytogenes*, were based on 24 primary studies with 220 observations [24,25,26,27,28,29,30,31,32,33,34,35,36,37,38,39,40,41,42,43,44,45,46,47], *Salmonella* spp. based on 14 primary studies with 100 observations [26,30,32,33,34,35,37,48,49,50,51,52,53,54], and *S. aureus* based on 25 studies with 190 observations [24,25,26,28,29,30,32,33,35,37,38,43,46,48,49,50,51,52,55,56,57,58,59,60,61].

Overall, the majority of LAB sources were cheese. For assays of *L. monocytogenes*, 92.65% of LAB were isolated from cheese, with the remainder sourced from milk (3.6%), mixed dairy products (3.2%), and yogurt (0.55%). The majority of milk species were from cows (52.7%), followed by goats (1.81%), mixed sources (16.36%), sheep (17.32%), buffalo (0.45%), and unspecified sources (11.36%). 

Similarly, the LAB tested against *Salmonella* spp. mainly originated from cheese (91%), milk (3.4%), and mixed dairy products (5.6%), of which 46.06% of milk species were from cow’s milk, with the remainder from goat (8.98%), mixed sources (6.74%), sheep (16.85%), buffalo (3.37%), or unspecified sources (18%). 

For *S. aureus*, 70.32% of tested LAB were from cheese, with the remaining cases originating from milk (15.38%), mixed dairy products (13.18%), and yogurt (1.12%), with the milk sources coming from cow’s milk (35.71%), goat (2.75%), mixed (21.98%), sheep (23.63%), buffalo (1.65%), or unspecified sources (14.28%).

In this study, the main genera of LAB obtained were *Lactobacillus* (n = 310), *Pediococcus* (n = 30), *Enterococcus* (n = 104), *Lactococcus* (n = 34), *Lacticaseibacillus* (n = 19) and *Leuconostoc* (n = 13). In terms of the types of data analyzed, there were no significant differences in the observed ID between the supernatant (n = 476) and bacteriocin (n = 37), leading to the decision to combine the data for analysis. 

### 3.2. Overarching Meta-Regression Models

The outcomes of the overarching meta-regression models are shown in Table 1. First of all, these results demonstrate that, overall, *L. monocytogenes* presented greater susceptibility to the LAB supernatant (i.e., higher inhibition values) compared to *Salmonella* spp., and *S. aureus*, although there were no differences between the latter two. Table 1 shows the pooled outcome of the LAB genera separated by type of pathogen. There, it can be observed that *Enterococcus* strains (15.90 ± 2.138 mm) were in general linked to higher inhibition values against *L. monocytogenes*, followed by *Lacticaseibacillus* (13.95 ± 2.313 mm), *Lactococcus* (13.12 ± 2.129 mm), *Lactobacillus* (11.96 ± 1.886 mm), *Leuconostoc* (10.25 ± 2.712 mm) and *Pediococcus* (8.735 ± 4.663 mm). 

Regarding *Salmonella* spp., *Enterococcus* (12.00 ± 1.195 mm), *Lactobacillus* (12.36 ± 1.148 mm), and *Lactococcus* (12.76 ± 1.194 mm) had no significant differences (*p* > 0.05) between their inhibition values, although *Lactococcus* had numerically higher inhibition diameters than the other two.

With regards to *S. aureus*, the LAB genus which displayed higher inhibition were *Enterococcus* (11.01 ± 2.105 mm), *Lacticaseibacillus* (11.89 ± 0.573 mm), *Lactobacillus* (11.35 ± 1.096 mm), and *Lactococcus* (11.33 ± 9.578 mm), followed by *Leuconostoc* (7.173 ± 0.287 mm) and *Pediococcus* (7.173 ± 0.225 mm). An overall higher inhibition diameter against this pathogen was obtained by *Lacticaseibacillus*.

The pooled IDs were also estimated using the susceptibility test method from the overarching model, and it was found that, as a whole, the well diffusion test method produced higher inhibition halos than those produced by the spot and disk diffusion tests (Table 1). There were a few exceptions, specifically, in the cases of *Lactobacillus* against *L. monocytogenes*, and *Lactobacillus* against *S. aureus*., for which spot and disk tests were found to be more closely associated with higher inhibition levels.

### 3.3. Pathogen-Specific Meta-Regression Models

#### 3.3.1. *Listeria monocytogenes*

The best-fit regression model for *L. monocytogenes* (Table 2) included the LAB genus, pathogen concentration, and incubation time. The *Enterococcus* supernatant provided overall higher inhibition of the pathogen; meanwhile, *Leuconostoc* and *Lactobacillus* supernatants were found to provide significantly lower inhibition, which can be deduced from the higher intercept for ID. 

**Figure 2:**
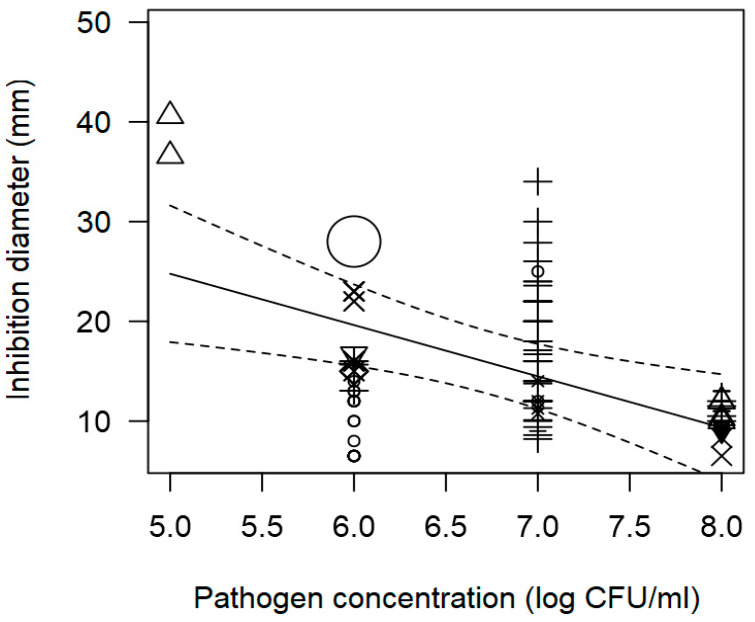
Scatter plot depicting the effect (*p* = 0.006) of *L. monocytogenes* concentration on the inhibition diameters. Markers symbolize bacterium: ○ = *Enterococcus*, ∆ = *Lacticaseibacillus*, + = *Lactobacillus*, × = *Lactococcus*, ◊ = *Leuconostoc*, ∇ = *Pediococcus*; and marker size is proportional to the study size. Solid line represents fitted values and dashed lines the 95% confidence interval.

**Figure 3:**
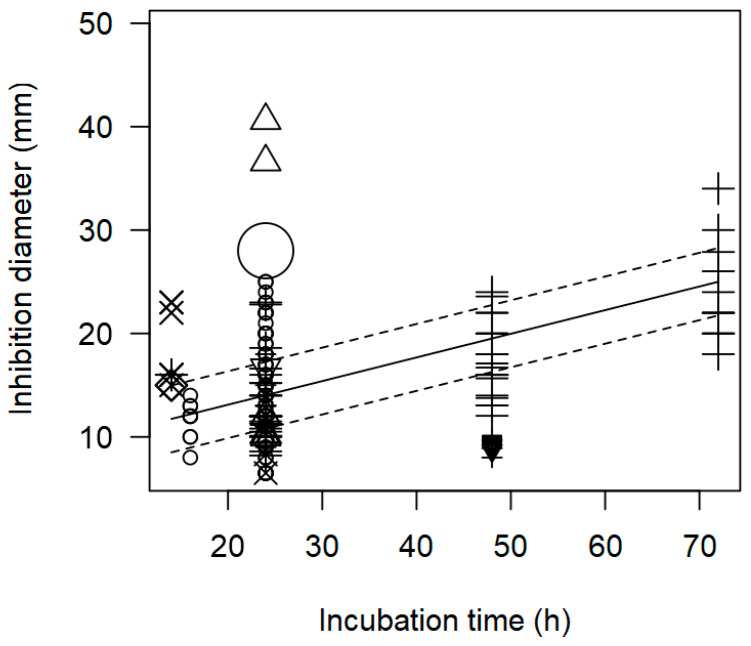
Scatter plot depicting the effect (*p* < 0.001) of incubation time on the inhibition diameters. Markers symbolize bacterium: ○ = *Enterococcus*, ∆= *Lacticaseibacillus*, + = *Lactobacillus*, × = *Lactococcus*, ◊ = *Leuconostoc*, ∇ = *Pediococcus*; and marker size is proportional to the study size. Solid line represents fitted values and dashed lines the 95% confidence interval.

The meta-regression model (Figure 2 and Figure 3) also demonstrated a significant inverse association between the concentration of pathogen inoculated in the agar and the measured ID, for instance, at a pathogen concentration of 8 log CFU/mL, an ID value of ~12 mm was measured. A direct association between incubation time and observed ID was also found (Table 2); at longer incubation times, for instance, 72 h, the inhibition values measured tended to be higher than 20 mm.

**Figure 4:**
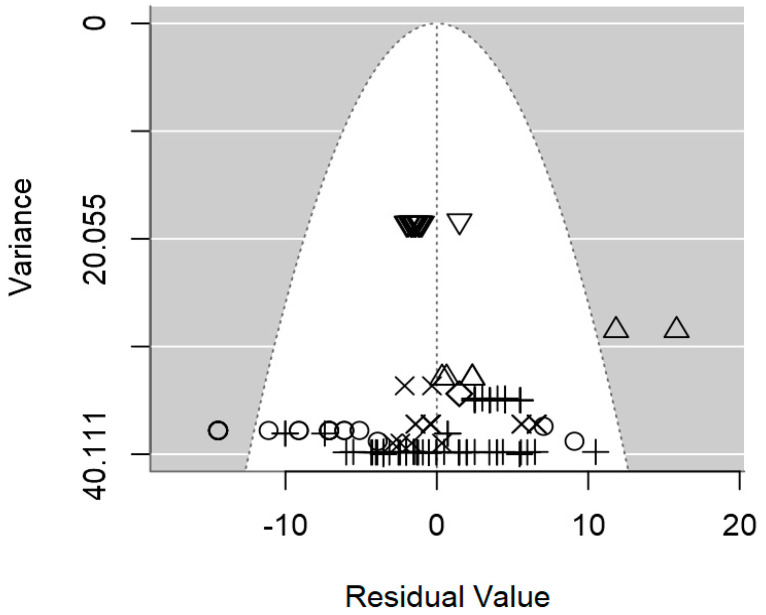
Funnel plot of the meta-regression model of the inhibition diameter produced by lactic acid bacteria supernatant against *L. monocytogenes*. Markers symbolize bacterium: ○ = *Enterococcus*, ∆ = *Lacticaseibacillus*, + = *Lactobacillus*, × = *Lactococcus*, ◊ = *Leuconostoc*, ∇ = *Pediococcus*.

In terms of publication bias, a symmetrical distribution of data points could be observed within the funnel (Figure 4), which provides no discernible evidence of publication bias. Likewise, the *p*-value for the presence of publication bias was not significant (*p* = 0.977). Heterogeneity analysis pointed out a moderate intra-class correlation (I^2^ = 55.1%), from which 33.8% could be explained jointly by the three significant moderators. It is likely that the highly unbalanced nature of the data, which hindered the inclusion of other moderators was the cause of the relatively low R^2^. 

#### 3.3.2. *Salmonella* spp.

The meta-regression model fitted to the ID outcomes for *Salmonella* spp. allowed the assessment of the effects of the susceptibility method, type of agar, and incubation time. Results (Table 3) showed that the well diffusion method and the Brain Heart Infusion (BHI) agar resulted in the highest inhibition diameters measured. Conversely, the disk diffusion method and de Man Rogosa and Sharpe (MRS) agar were significantly correlated with lower values of inhibition diameter (mm).

**Figure 5:**
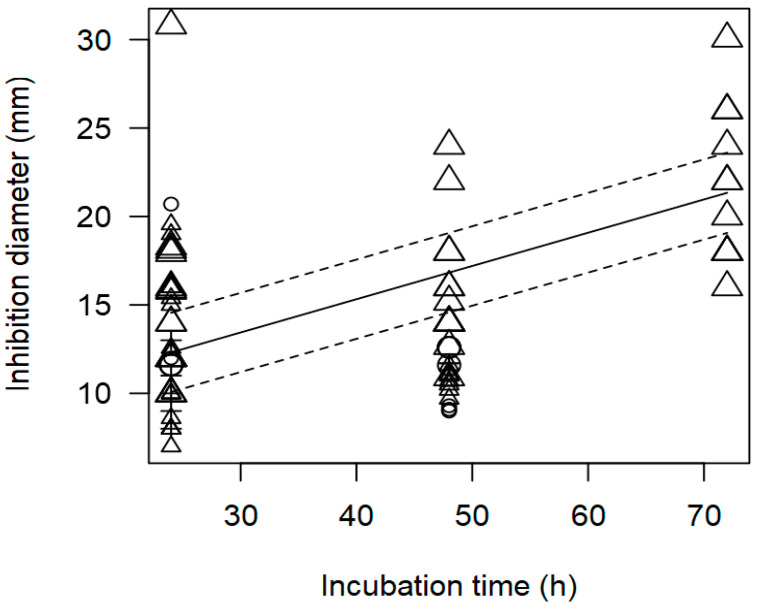
Scatter plot depicting the effect (*p* < 0.001) of incubation time on the inhibition diameters of *Salmonella* spp. Markers symbolize bacterium: ○ = *Enterococcus*, ∆ = *Lactobacillus*, + = *Lactococcus*; and marker size is proportional to the study size. Solid line represents fitted values and dashed lines the 95% confidence interval.

As found with the meta-regression for *L. monocytogenes*, the model of inhibition against *Salmonella* spp. showed a positive relationship between incubation time and measured ID values (*p* < 0.001). Figure 5 shows the data scattered at three incubation times, 24, 48, and 72 h, and it is mainly *Lactobacillus* strains that were associated with higher inhibition diameters at longer incubation times.

**Figure 6:**
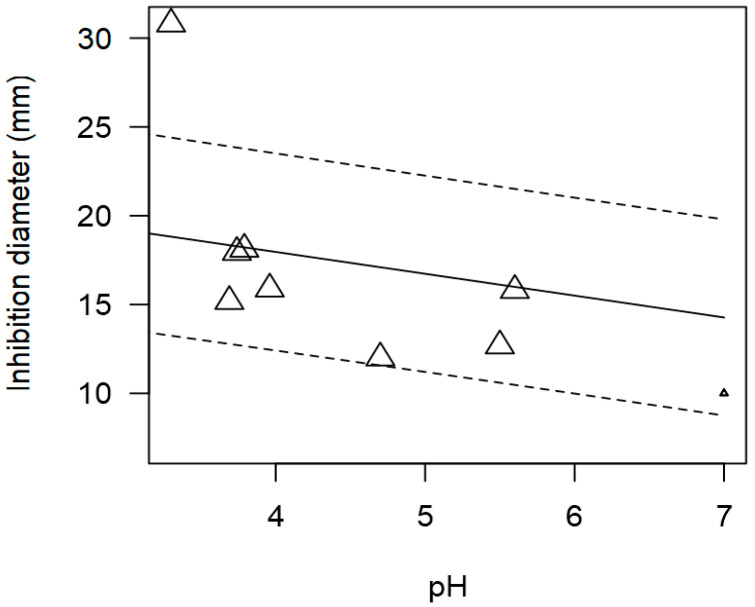
Scatter plot depicting the effect (*p* < 0.001) of pH on the inhibition diameters of *Lactobacillus* against *Salmonella* spp. Marker size is proportional to study size. The pH was not added to the final model as a moderator because there were too few incidences. Solid line represents fitted values and dashed lines the 95% confidence interval.

Although pH could not be included as a moderator in the meta-regression model due to its very low incidence, the relationship between pH and ID values was still explored. The tendency of pH of the supernatant from LAB and inhibition diameter values were significantly correlated (*p* < 0.001). Figure 6 shows that *Lactobacillus* displayed higher inhibitory activity at a more acidic pH compared to a neutral pH. 

**Figure 7:**
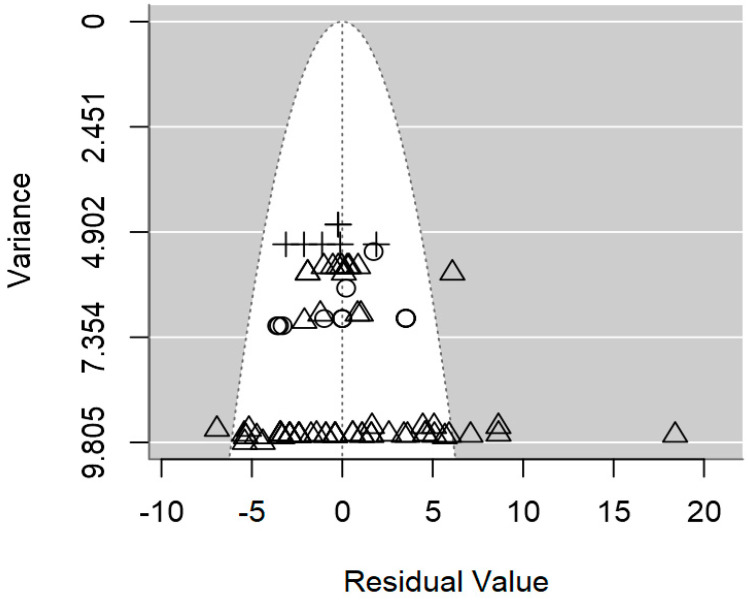
Funnel plot of the meta-regression model of the inhibition diameter produced by lactic acid bacteria supernatant against *Salmonella* spp. Markers symbolize bacterium: ○ = *Enterococcus*, ∆ = *Lactobacillus*, + = *Lactococcus*.

The publication bias *p*-value of the meta-regression on ID values for *Salmonella* spp. was not significant (*p* = 0.217; Table 3), which was also supported by the symmetrical dispersion of the data points of the funnel plot shown in Figure 7. The intra-class correlation of this meta-regression was fairly low (I^2^ = 33.6%), and the three moderators were able to explain 52.3% of the between-study variability (Table 3).

#### 3.3.3. *Staphylococcus aureus*

The data-driven model fitted for the pathogen *S. aureus*, included LAB genus, pathogen concentration, incubation time, and assay method as significant moderators. The results compiled in Table 4 show that higher measurements of inhibition diameter were associated with the well diffusion method, and mainly by *Enterococcus* strains. In contrast, the disk diffusion method led to significantly lower inhibition values, as did LAB strains belonging to the genera *Leuconostoc* and *Pediococcus*. 

In this model, the intra-class correlation was relatively low (I^2^ = 38.8%), and the four moderators could explain only 19.8% of the total between-study variability (Table 4). Since the variance associated with the different studies is not high, the unexplained part of the variance could be attributed to, more likely, “false” non-significant moderators arising from the data’s lack of balance or, less likely, to traits not measured in this study.

**Figure 8:**
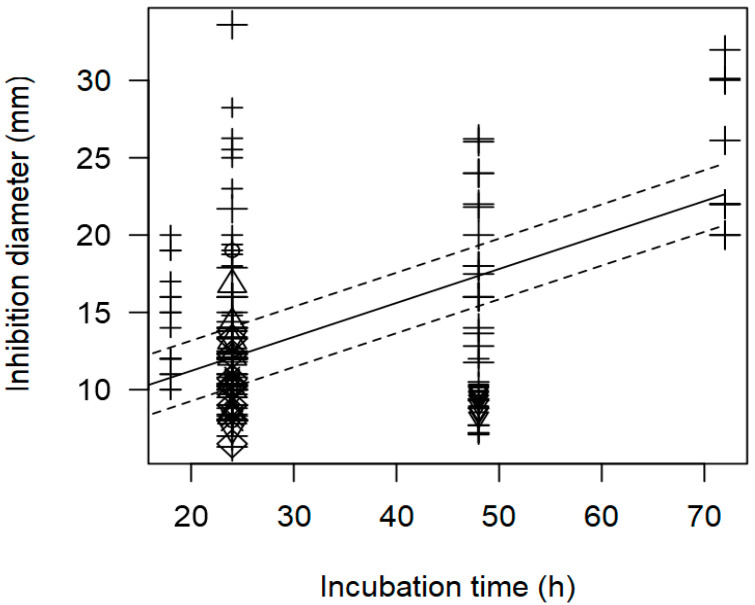
Scatter plot depicting the effect (*p* < 0.001) of incubation time on the inhibition diameters. Markers symbolize bacterium: ○ = *Enterococcus*, ∆ = *Lacticaseibacillus*, + = *Lactobacillus*, × = *Lactococcus*, ◊ = *Leuconostoc*, ∇ = *Pediococcus*; and marker size is proportional to the study size. Solid line represents fitted values and dashed lines the 95% confidence interval.

In addition, the meta-regression model showed a significant effect of the incubation time (h) on the inhibition diameter of the indicator strains. The longer the incubation period of the indicator strain with the tested supernatant, the greater the inhibition diameter. In Figure 8, a significant effect of incubation time (*p* < 0.001) on the inhibition of *S. aureus* is clearly shown. In this case, *Lactobacilli* were associated with higher inhibition diameters (>20 mm) when the plates were incubated for a longer time (72 h).

**Figure 9:**
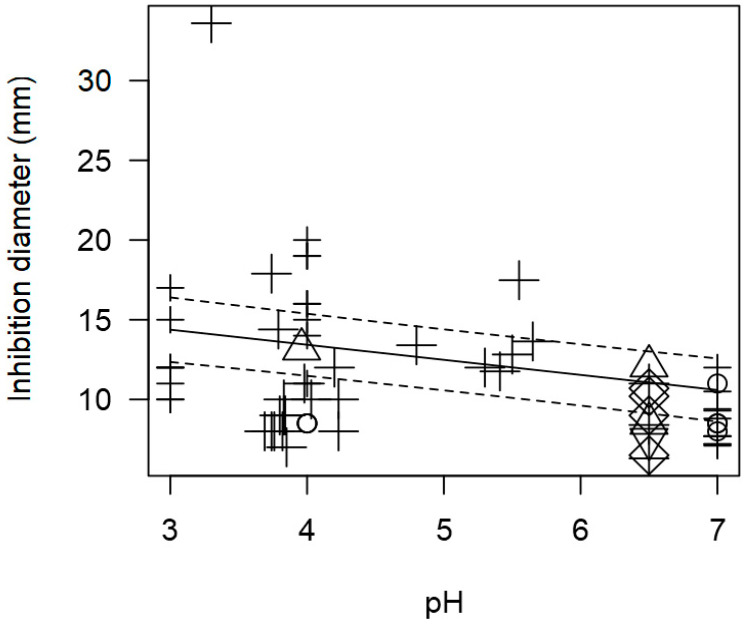
Scatter plot depicting the effect (*p* < 0.001) of pH on the inhibition diameters. Markers symbolize bacterium: ○ = *Enterococcus*, ∆ = *Lacticaseibacillus*, + = *Lactobacillus*, × = *Lactococcus*, ◊ = *Leuconostoc*, ∇ = *Pediococcus*; and marker size is proportional to study size. pH was not added in the final model as a moderator because there were too few incidences. Solid line represents fitted values and dashed lines the 95% confidence interval.

Once again, due to the few outcomes with information on pH, this variable could not be included in the full meta-regression model. Nonetheless, the association between pH and inhibition diameter was assessed to find a significant inverse correlation (*p* < 0.001). Figure 9 shows that more acidic pH values (3 to 5) would tend to increase the measured inhibition diameters, resulting in higher values (approximately higher than 12 mm). On the other hand, a more neutral pH (6 to 7) results in less inhibition with lower diameter values (≤10 mm). 

**Figure 10:**
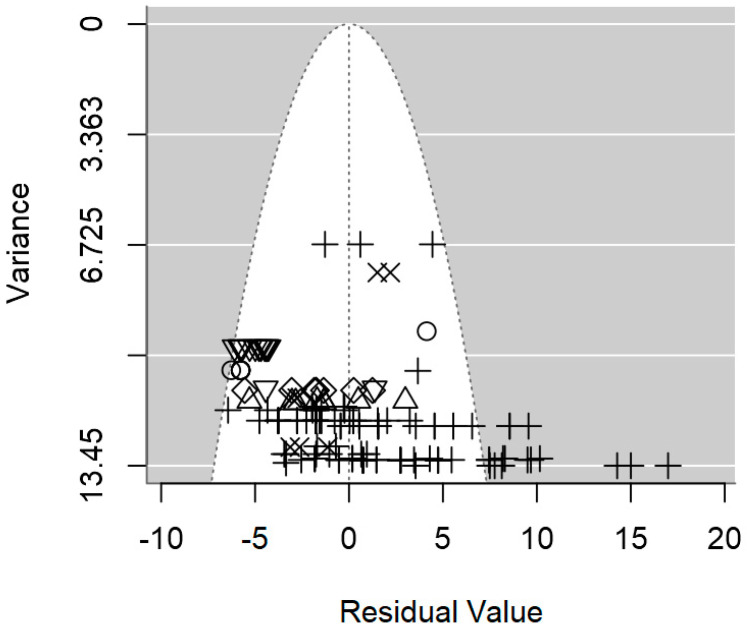
Funnel plot of the meta-regression model of the inhibition diameter produced by lactic acid bacteria supernatant against *S. aureus*. Markers symbolize bacterium: ○ = *Enterococcus*, ∆ = *Lacticaseibacillus*, + = *Lactobacillus*, × = *Lactococcus*, ◊ = *Leuconostoc*, ∇ = *Pediococcus*.

In Figure 10, the symmetrical distribution of the data in the plots indicates the absence of significant publication bias. Moreover, these plots imply minimal residual heterogeneity among the studies (τ^2^_res_), with no discernible evidence of publication bias (*p* = 0.492).

## 4. Discussion 

Indigenous starter cultures have become a trend in the dairy food industry due to their fast adaptation to the cheese microenvironment and their ability to provide traditional cheese with its characteristic organoleptic features [4]. Starter LAB inhibit pathogenic or spoilage microorganisms by competition or ecosystem conditioning, such as lowering the pH and reducing water activity [62]. LAB produce several important enzymes, including proteases, lipases, bacteriocins, glucoamylases, peptidoglycan hydrolases, ureases, and phenol oxidases, which can also be found in supernatants [63].

In this meta-analysis, we found that most studies assessing the antimicrobial activity of LAB originated from cheese, especially from cow milk sources. A notable limitation found in this study was the lack of species-level insights due to insufficient statistical power, which restricted findings at the genus level. Additionally, the composition of the supernatant used in the experiments was not described in detail by the original authors, which hampers an in-depth analysis as to the source of inhibition. However, a significant advantage was identifying the most common LAB naturally occurring in dairy matrices, which are likely to be more effective against pathogens in food environments.

The most common LAB were *Lactobacillus*, *Enterococcus*, and *Lactococcus*, in accordance with previous research [4,63]. In a meta-analysis of traditional artisanal Irish cheese microbiomes, LAB were found to have a prevalence of 91.3%, with *Lactococcus lactis*, *Streptococcus thermophilus*, and *Lactobacillus helveticus* being the most prevalent species. These LAB harbored genes encoding for class II/III bacteriocins and metalloproteases [64].

Bacteriocins are small peptides synthesized in the ribosome and are generally considered safe for consumption as they are degraded by gastric and pancreatic enzymes during digestion due to their chemical nature, although some may present toxicity at high concentrations [63]. Currently, the use of bacteriocins as food additives for dairy production is limited. In Europe, nisin (E 234), synthesized by *Lactococcus lactis*, is approved by the European Food Safety Authority (EFSA) [2]. Nisin was found to be effective against *L. monocytogenes* in dairy products, reducing its presence by up to 1 log cycle in cottage cheese stored at 20 °C for 7 days at a pH of 4.6–4.7 and exhibits antimicrobial activity against numerous other Gram-positive bacteria, including *Staphylococcus*, *Bacillus*, and *Clostridium* [65,66,67].

Table 1 shows the conditions normalized to 24 h of incubation with a pathogen at 7 log CFU/mL. Contrasting the effects of LAB supernatant on different pathogens, *L. monocytogenes* showed greater susceptibility compared to *Salmonella* spp. and *S. aureus*, especially when tested against *Enterococcus*. Enterococci LAB, commonly found in the gastrointestinal tracts of humans and dairy animals, can play both beneficial and harmful roles in milk and dairy products [68].

The best-fit regression model for *L. monocytogenes* explained 33.8% of the total variation and showed a significant inverse association between pathogen concentration and inhibition diameter (ID). A meta-analysis of in situ pathogen inhibition in cheese found a similar negative association between inoculum and log reduction (*p* = 0.025), indicating lower inhibitory effects with higher pathogen populations [68].

Within *Salmonella* spp., *Lactococcus* showed the highest inhibition values. Contaminated raw milk or curd can lead to *Salmonella* surviving the fermentation and ripening processes, resulting in contaminated final products [69]. The meta-regression model for *Salmonella* spp. ID outcomes accounted for 52.3% of the variability, showing a positive relationship between the incubation time and ID values (*p* < 0.001). This is in accordance with previous studies showing that while LAB inoculum exerts immediate antimicrobial effects on the indicator strain, the supernatant shows a greater effect with longer incubation [68].

Additionally, a significant correlation was found between the pH of LAB supernatants and ID values, with higher IDs associated with acidic pH. Different composition of organic acids in supernatants result in varying pH levels. Neutralizing the pH standardizes the testing conditions, ensuring that the observed effects are due to antimicrobial properties rather than pH changes, allowing for consistent comparisons [63].

*S. aureus* in contaminated milk and dairy products is a significant food safety concern, this pathogen is often introduced during milking due to poor hygiene or contaminated equipment [70]. *S. aureus* was highly inhibited by *Lacticaseibacillus* and the significant moderators for the final inhibition included the LAB genus, pathogen concentration, incubation time, and assay method, accounting for 19.8% of the variation. As with *L. monocytogenes*, longer incubation resulted in higher IDs, with acidic pH values increasing IDs. Furthermore, *S. aureus* is more susceptible to the effect of organic acids [66,67,68,69,71].

The pooled IDs showed that the well diffusion test method produced higher inhibition halos than the disk tests, likely due to superior diffusion in wells versus disks. Susceptibility methods vary in antimicrobial applications: directly (agar spot), via a disk (disk diffusion), or through a well (well diffusion) [66].

## 5. Conclusions

In conclusion, this meta-analysis has revealed that pathogen concentration negatively influences the effect of the supernatant; the longer the incubation time, the better the results of inhibition, and the more acidic the supernatant pH, the higher the inhibition; however, there is an issue regarding the reporting of pH measurements for supernatant screening assays. The more adequate LAB to be used as biological control agent changes according to the specific pathogen.

Future research should detail the components of the LAB supernatant, investigate the impact of uncultured organisms on LAB antimicrobial activity, and explore the specific supernatant composition. Increasing the statistical power of species-level analysis could provide more detailed insights into LAB effectiveness. By understanding the dynamics of autochthonous LAB species and their antimicrobial activity against foodborne pathogens, stakeholders can implement targeted interventions to improve the safety and quality of dairy products and ultimately safeguard public health. Continued research on the microbiological quality of cheeses, including the exploration of bacteriocins produced by dairy strains, is essential for enhancing food safety and expanding the range of functional dairy products. 

## Figures and Tables

**Table 1:** Pooled inhibition diameters (mean and standard error in mm) produced by lactic acid bacteria (LAB) supernatant using pathogen’s concentration of 7.0 log CFU/mL and an incubation time of 24 h, as estimated by meta-regression models adjusted by foodborne pathogen. The number of observations (n) and number of primary studies (N) are shown.

Pathogen ^1^	LAB Genus	Method	Pooled Inhibition Diameter ^2,3^ (SE) [mm]		n	N
*Listeria monocytogenes* ^A^	*Enterococcus*	Overall	15.90 ^A^ (2.138)		38	24
		Well		19.47 ^a^ (2.409)	28	
		Disk		10.98 ^c^ (1.962)	10	
	*Lacticaseibacillus*	Overall	13.95 ^B^ (2.313)		8	
		Well		28.57 ^a^ (5.058)	4	
		Disk		11.73 ^c^ (1.835)	4	
	*Lactobacillus*	Overall	11.96 ^C^ (1.886)		92	
		Spot		14.87 ^b^ (2.883)	14	
		Well		12.02 ^c^ (2.541)	18	
		Disk		10.88 ^c^ (1.598)	60	
	*Lactococcus*	Overall	13.12 ^B^ (2.129)		20	
		Spot		17.75 ^a^ (2.076)	14	
		Disk		10.23 ^c^ (1.792)	6	
	*Leuconostoc*	Overall	10.25 ^C^ (2.712)		5	
		Spot		14.04 ^b^ (3.045)	4	
	*Pediococcus*	Overall	8.735 ^C^ (4.663)		14	
		Well		8.800 ^c^ (3.025)	14	
*Salmonella* spp ^B^	*Enterococcus*	Overall	12.00 ^A^ (1.195)		13	14
		Well		13.39 ^a^ (1.408)	12	
	*Lactobacillus*	Overall	12.36 ^A^ (1.148)		69	
		Well		13.58 ^a^ (1.362)	18	
		Disk		11.13 ^b^ (1.361)	51	
	*Lactococcus*	Overall	12.76 ^A^ (1.194)		7	
		Disk		10.82 ^b^ (1.433)	5	
*Staphylococcus aureus* ^B^	*Enterococcus*	Overall	11.01 ^A^ (2.105)		10	25
		Well		14.16 ^a^ (0.716)	4	
		Disk		5.28 ^d^ (0.366)	6	
	*Lacticaseibacillus*	Overall	11.89 ^A^ (0.573)		9	
		Well		12.36 ^b^ (1.501)	5	
		Disk		12.00 ^b^ (1.197)	4	
	*Lactobacillus*	Overall	11.35 ^A^ (1.096)		133	
		Well		10.00 ^c^ (1.772)	67	
		Disk		12.03 ^b^ (1.394)	66	
	*Lactococcus*	Overall	11.33 ^A^ (9.578)		7	
		Disk		11.24 ^b^ (0.529)	5	
	*Leuconostoc*	Overall	7.173 ^B^ (0.287)		8	
		Well		7.198 (0.304)	8	
	*Pediococcus*	Overall	7.173 ^B^ (0.225)		15	
		Well		7.161 (0.232)	15	

^1^ Different superscript uppercase letters indicate significant differences in the pooled inhibition diameter between pathogens according to a single meta-regression including pathogen, lactic acid bacteria (LAB) genus, susceptibility test method, aliquot volume, pathogen concentration, and incubation time as moderators. ^2^ Different superscript uppercase letters indicate significant differences in the pooled inhibition diameter between LAB genera according to three meta-regression models (by pathogen) including LAB genus, aliquot volume, pathogen concentration and incubation time as moderators. ^3^ Different superscript lowercase letters indicate significant differences in the pooled inhibition diameter between LAB genera according to three meta-regression models (by pathogen), including LAB genus, aliquot volume, pathogen concentration, incubation time, and susceptibility test method as moderators.

**Table 2:** Final meta-regression model of inhibition diameter produced by lactic acid bacteria (LAB) supernatant against *L. monocytogenes* as a function of LAB genus, pathogen concentration (log CFU/mL), and incubation time (h). The number of observations (n) per LAB genus, heterogeneity analysis, and *p*-values for the publication bias test are shown.

Parameter	Estimate	Standard Error	*p*-Value	n	Heterogeneity Analysis ^1^
Intercept	45.67	12.41	0.002		s^2^ = 53.0
LAB genus					τ^2^ = 65.2
* Lacticaseibacillus*	−1.217	2.112	0.565	8	τ^2^_res_ = 43.1
* Lactobacillus*	−3.375	1.580	0.033	92	R^2^ = 33.8%
* Lactococcus*	−2.248	1.787	0.208	20	I^2^ = 55.1%
* Leuconostoc*	−5.140	2.528	0.042	5	
* Enterococcus*	-	-	-	81	
Pathogen concentration	−5.037	1.840	0.006		Publication bias
Incubation time	0.229	0.229	<0.0001		*p* = 0.977

^1^ Heterogeneity analysis encompasses within-study variability (s^2^), between-study variability (τ^2^), and intra-class correlation (I^2^) of the null model, and residual between-study variability (τ^2^_res_), and between-study variability explained by significant moderators (R^2^) from the full model.

**Table 3:** Final meta-regression model of inhibition diameter produced by lactic acid bacteria (LAB) supernatant against *Salmonella* spp. as a function of susceptibility test method, agar, and incubation time (h). Heterogeneity analysis and p-value of the publication bias test are shown.

Parameter	Estimate	Standard Error	*p*-Value	n	Heterogeneity Analysis ^1^
Intercept	10.41	2.250	<0.0001		
Susceptibility method					
Disk diffusion	−2.529	0.979	0.009		s^2^ = 25.1
Agar					τ^2^ = 12.8
MH	−1.540	2.920	0.589	11	I^2^ = 33.6%
MRS	−8.114	2.273	0.001	20	τ^2^_res_ = 6.1
Nutrient	3.575	2.553	0.161	5	R^2^ = 52.3%
TSA	−2.111	2.037	0.300	10	
BHI	-	-	-	43	Publication bias
Incubation time	0.188	0.005	<0.0001		*p* = 0.217

^1^ Heterogeneity analysis encompasses within-study variability (s^2^), between-study variability (τ^2^), and intra-class correlation (I^2^) of the null model, and residual between-study variability (τ^2^_res_), and between-study variability explained by significant moderators (R^2^) from the full model. MH: Mueller-Hinton; MRS: de Man Rogosa and Sharpe; TSA: Trypticase Soy Agar; BHI: Brain Heart Infusion.

**Table 4:** Final meta-regression model of inhibition diameter produced by lactic acid bacteria (LAB) supernatant against *S. aureus*, as a function of LAB genus, pathogen concentration (log CFU/mL), incubation time (h), and susceptibility test method. Heterogeneity analysis and *p*-value of the publication bias test are shown.

Parameter	Estimate	Standard Error	*p*-Value	n	Heterogeneity Analysis ^1^
Intercept	22.04	2.545	<0.0001		
LAB genus					s^2^ = 31.8
* Lacticaseibacillus*	−0.923	1.481	0.533	9	τ^2^ = 20.2
* Lactobacillus*	−1.295	1.208	0.283	133	I^2^ = 38.8%
* Lactococcus*	−1.758	1.842	0.340	7	τ^2^_res_ = 16.2
* Leuconostoc*	−5.666	1.835	0.002	8	R^2^ = 19.8%
* Pediococcus*	−7.335	2.154	0.001	15	
* Enterococcus*	-	-	-	10	
Pathogen concentration	−1.888	0.297	<0.0001		Publication bias
Incubation time	0.220	0.005	<0.0001		*p* = 0.492
Susceptibility method					
Disk diffusion	−2.373	0.483	<0.0001		

^1^ Heterogeneity analysis encompasses within-study variability (s^2^), between-study variability (τ^2^), and intra-class correlation (I^2^) of the null model, and residual between-study variability (τ^2^_res_), and between-study variability explained by significant moderators (R^2^) from the full model.

## Data Availability

The original contributions presented in the study are included in the article, further inquiries can be directed to the corresponding author.
